# Experience of adverse events with cerebral propofol testing in patients with drug resistant epilepsy

**DOI:** 10.1038/s41598-018-36031-w

**Published:** 2019-01-24

**Authors:** Marta Szantroch, Aleksandra Bala, Andrzej Rysz, Jarosław Żyłkowski, Andrzej Marchel

**Affiliations:** 1 Department of Neurosurgery, Medical University of Warsaw, Banacha 1a, 02-097 Warsaw, Poland; 2 Faculty of Psychology, University of Warsaw, Poland, Stawki 5/7, 00-183 Warsaw, Poland; 3 Second Department of Radiology, Medical University of Warsaw, Banacha 1a, 02-097 Warsaw, Poland

## Abstract

The aim of this study was to assess the type and frequency of adverse events during the Wada test conducted with propofol as an anaesthetic agent. In total, 122 patients with temporal lobe epilepsy underwent the Wada test with propofol between 2006 and 2016 as part of presurgical evaluation at the Department of Neurosurgery of the Medical University of Warsaw. The Wada test was conducted bilaterally on 118 patients (236 cases). In four cases, due to complications, the test was conducted only unilaterally, which resulted in a total of 240 cases. Those cases were further analysed for the presence of adverse events. In all cases, intracranial circulation angiography (via the transfemoral approach) was performed before memory and language testing. Of the 122 patients, adverse events were observed in 75 patients (61.4%). Serious complications were notably rare and observed only in two patients (1.6%): one patient had a carotid artery dissection, and the other had a pseudoaneurysm at the puncture site. Mild adverse events (e.g., shivers or pain of the eye) were highly common – we observed them in 71 patients (58%), but they were short-term and well-tolerated by the subjects. Two patients (1.6%) had a seizure during the Wada test. Most of the adverse events occurring during the Wada test with propofol were mild and short-lived. Considering a small risk of serious damage to health, this procedure can be perceived as a good method for assessing language and memory in a fraction of the epilepsy surgery candidates.

## Introduction

In a number of centres, the Wada test is still considered to be the gold standard for presurgical assessment of language and memory functions in patients with medically intractable temporal lobe epilepsy. Despite the development of neuroimaging methods (mostly functional magnetic resonance imaging) for assessing these functions^1,2^, the employment of the Wada test, as part of preoperative evaluation of candidates for neurosurgical treatment of epilepsy, has many indications. For example, there is a need for better control of actions of low-cooperating patients or those with a reduced level of functioning (who may not sufficiently focus on performing tasks in fMRI)^3,4^ or better visualization of the possible effects of post-surgical lesions.

The Wada test requires applying a transfemoral injection of a fast-acting anaesthetic agent to the internal carotid artery to invoke a transient inhibition of the ipsilateral cerebral hemisphere. The first anaesthetic used for this procedure was sodium amobarbital (amythal)^5^. With availability problems worldwide, various short-acting intravenous (IV) anaesthetic agents have been tested as an alternative to amythal^6–11^. Propofol, first described as an anaesthetic drug in the Wada test by Bazin^12^, has been indicated as a safe option for the Wada test in many studies^13–18^, but it is not free from adverse events (AEs). Several researchers have stated that since the Wada test is an invasive method, it might have serious neurological complications^19–21^; therefore, its use should be considered seriously.

In this paper, we report our experience with the Wada procedure with propofol. We analyse the frequency and possible prognostic factors for the occurrence of AEs.

## Results

### AE distribution

In total, AEs occurred in 75 of 122 patients (61.4%). In 60 patients, adverse events during the Wada procedure occurred unilaterally and in 15 patients bilaterally, which resulted in 90 (37.5%) cases of AEs. Serious complications (Group 1) were very rare and observed in only two patients (1.6%): one patient had a carotid artery dissection (which caused a small area of ischaemia in the left frontal lobe and transient aphasia), and the other had a pseudoaneurysm at the puncture site. In both patients, the symptoms fully passed in a few months. Mild, short-lasting and transient AEs (Group 2) were observed in 71 patients (58%) and 86 cases (35.8%), respectively, and they included symptoms that might temporarily preclude logical contact and slightly postpone neuropsychological evaluation. AEs from this group were divided into several subgroups. The first (Group 2a) was composed of somatic symptoms: eye pain and/or lacrimation, which were very common and occurred in 40 of our patients (32%), and shivers, which occurred in 28 patients (22.9%), mostly immediately after the injection of the propofol. The second subgroup (Group 2b) included behavioural abnormalities, such as verbal disinhibition, viscosity, apathy and drowsiness, which was observed in 15 patients (12.2%), and the third subgroup (Group 2c) included emotional lability, such as sadness or uncontrollable laughter, which pertained to 19 patients (15.5%). In certain cases, patients had to be reminded that they needed to cooperate. All of the transient AEs passed within a few minutes after administering the drug. None of the assessments were invalid due to this type of AE. In the third group of AEs (epileptic seizures), we counted two such events (1.6%). In both cases, patients were agitated and confused and it was impossible to proceed with the Wada test. More details can be found in Tables 1 and 2. It should be noted that the sum of the symptoms (n = 102) presented in Table 2 does not match the number of patients (n = 71) or number of cases (halves of the procedure, n = 86) with AEs from Group 2. This finding is observed because a number of patients had more than one symptom. If the given symptom occurred bilaterally (twice), we counted it as one type of AE in a particular patient.

**Table 1:** Percentage of patients experiencing particular AEs.

	Number of patients (total n = 122)
All adverse events	75 (61.4%)
Adverse events Group 1	2 patients (1.6%)
Adverse events Group 2 occurring unilaterally/bilaterally*	56 patients (45.9%)/15 patients (12%)
Adverse events Group 3	2 patients (1.6%)
No adverse events	47 patients (38.5%)

*Unilaterally – in a particular patient AEs were present only during propofol injection into one hemisphere; bilaterally - AEs were present during propofol injection into both hemispheres.

**Table 2:** Distribution of symptoms of AEs from Group 2.

Symptoms		Number of patients with the occurrence of the symptom
Somatic symptoms - Group 2a	Eye pain & lacrimation	40 (32%)
	Shivers	28 (22.9%)
Behavioural abnormalities-Group 2b	Apathy & drowsiness	7 (5.7%)
	Verbal disinhibition & viscosity	8 (6.5%)
Emotional lability-Group 2c	Sadness	5 (4%)
	Laughter	14 (11.4%)

To allow comparisons between studies and to ensure greater clarity, we decided to report our results also using the widely known classification of Mikuni^17^. The complications of grade 1 were observed in 62 patients (50.8%); specifically, eye pain and lacrimation was observed in 40 patients, shivering in 28, and laughing and apathy in 17. We did not observe face contortion. The number of specific AEs do not sum up to 62 because a portion of patients had more than one type. Grade 2 adverse events in the form of a little confusion occurred in four patients (3.2%). No grade 3 side effects were noted in our study.

### Risk factors for AEs

We tried to determine if there were any differences in the occurrence of AEs with respect to group characteristics. Therefore, we used chi square analysis. No differences in frequency of AEs between males and females, and no relations with age, years of disease duration, age at epilepsy onset, as well as with presence of atypical anatomy of the vascular system were observed (Table 3).

**Table 3:** Cases of AEs in respect to demographic and clinical characteristics of patients.

	Left hemisphere (n = 72)			Right hemisphere (n = 50)		
	Number of AEs	Chi-Square; df	p value	Number of AEs	Chi-Square; df	p value
Sex (Male/Female)	32/29	0.22; 1	0.57	30/28	0.19; 1	0.71
Age in years (0–30/>30)	28/33	1.14; 1	0.29	26/32	1.73; 1	0.22
Years of the disease duration (0–20/>20)	37/25	3.56; 1	0.13	33/24	3.96; 1	0.36
Age at epilepsy onset in years (0–15/>15)	39/24	3.49; 1	0.06	30/26	2.58; 1	0.09
Atypical anatomy of the vascular system - fetal-type posterior circle of Willis: Yes (%)/No(%);	16 (58.5%)/45 (63.7%)	2.14	0.07	15 (60.3%)/43 (62.9%)	0.34; 1	0.23

Abbreviations: AEs = adverse events; Yes = number of AEs in patients with atypical vascular anatomy; No = number of AEs in group of patients without vascular anomalies.

Next, we verified if there was a greater incidence of AEs when a higher dose of propofol was administered (>10 mg versus 10 mg). We analysed cases, not patients, because in certain of the subjects, a higher dose was used bilaterally, and in others, the higher dose was only unilaterally. In the cases with the standard dose of propofol, 46% had complications (94 of 204 cases), whereas in the group with a higher dose of the anaesthetic, the percentage was considerably greater at 69.4% (25 of 36 cases).

We also looked for differences in the frequency of particular AEs with respect to the hemisphere of the propofol injection. Only one complication, uncontrolled laughter, differed statistically between left and right hemisphere propofol injection groups. The complication occurred far more often after propofol injection into the left hemisphere (Table 4). As can be seen, the sum of the symptoms in right (n = 60) and left hemisphere (n = 63) presented in Table 4 does not match the number of patients (n = 71) or number of cases (halves of the procedure, n = 86) with AEs from Group 2. A number of patients had more than one AE, and in certain of them, the same symptom occurred during both parts of the procedure (in two cases).

**Table 4:** Differences in AEs of Group 2 occurrence in respect to the side of the propofol injection.

		Injection to right hemisphere	Injection to left hemisphere	Chi-Square; df	p value
Somatic symptoms - Group 2a	Eye pain & lacrimation	26 (52%)	16 (22%)	2.38; 1	0.12
	Shivers	17 (34%)	17 (23%)	0.00; 1	1
Behavioural abnormalities-Group 2b	Apathy & drowsiness	4 (8%)	3 (4%)	0.14; 1	0.70
	Verbal disinhibition & viscosity	6 (12%)	7 (9%)	0.07; 1	0.78
Emotional lability-Group 2c	Sadness	2 (4%)	3 (4%)	0.20; 1	0.65
	Laughter	5 (10%)	17 (23%)	6.36; 1	0.01*

Abbreviations: AEs = adverse events; *p value significant

## Discussion

The Wada test is an invasive method that may carry a risk of potential neurological and non-neurological complications resulting from intra-arterial catheter insertion and/or the effect of the anaesthetic agent used for the procedure. In modern cerebral angiography, neurological complications resulting from the procedure are relatively rare and range from 0.3% to 2.3%^20–22^. The AEs of the procedure include stroke, transient global amnesia, thromboembolism, subarachnoid haemorrhage and arterial dissections. Additionally, several investigators have noted possible non-neurological complications, such as allergic cutaneous reactions, haematomas at the puncture site and nasal congestion, but their numbers vary across studies^21,22^.

Many studies have focused on comparing the effectiveness and risks of propofol employment in the Wada test as an alternative to sodium amobarbital due to problems with obtaining the latter drug. Takayama *et al*.^15^ analysed 67 right-handed patients who were undergoing the Wada procedure, 14 of whom were assessed for potential AEs of propofol. The authors observed laughing and head and eye version in three of the 14 examined patients (21%) directly after the injection, and face contortions and lacrimation in one patient. No other complications were noted. Mikuni *et al*.^17^ examined 58 patients with different neurological conditions (brain tumours, temporal/frontal lobe epilepsy, arteriovenous malformations), who were injected with propofol during the Wada test. The researchers divided the complications (according to their likelihood of causing Wada discontinuation or generating a false result) into three grades and assessed the percentage of AEs for each category. Grade 1 was composed of eye pain, shivering, face contortion, lacrimation, laughing and apathy, and it occurred in 10.3% of the patients (6/58). Grade 2 AEs included confusion, involuntary movement, and head and eye version in 10.3% of patients (6/58). Grade 3 AEs included increased muscle tone, twitching and rhythmic movements or tonic posture and occurred in seven patients (12%). In addition, Mikuni looked for the risk factors correlating with the occurrence of grade 3 AEs. Age older than 55 years, a total injection dose of propofol higher than 20 mg and a second injection dose of propofol greater than 10 mg were found to influence the risk of AEs. In another study, Alsallom^16^ observed AEs in four of nine patients who were included in the study. AEs were mostly transient; one patient experienced behavioural changes after administering propofol into both ICA, two experienced shivering, and one complained of eye and facial pain. In addition, this patient developed a transient segmental spasm of the ICA, but there were no major AEs observed. Mikati and colleagues^14^ compared a group of 25 patients who received a propofol injection during the Wada test with 15 patients who received amobarbital. The researchers reported one patient who experienced an episode of confusion and combativeness after receiving propofol. All of the patients who were injected with propofol had ipsilateral facial and ocular flushing and discomfort. Only occasional patients had chills and extended episodes of tremulousness, but this did not influence the examination process. Magee and collaborators^18^ also compared a group of patients injected with propofol to those injected with sodium amobarbital to compare the effectiveness of the former. The researchers used Mikuni’s^17^ categorizing system for AEs and found no significant differences between those two anaesthetic agents. The complications observed most often after the unilateral injection of propofol were those of grade 1 in 21.8% of the patients (eye pain, shivering, face contortion, lacrimation, apathy and laughing), and fewer had grade 2 AEs (only 7.1%). No grade 3 side effects were noted after propofol injection. Curot and colleagues^13^, using a modified Mikuni’s classification, also observed AEs in 26 of 58 propofol injections. Those occurring most often were disturbances of consciousness and ocular disorders. The others consisted of movement and dystonic disorders, pain, and mood and autonomic disorders. All of these events were transient and rather mild. In our research, complications of Mikuni’s grade 1 were observed in 50.8% of patients, and grade 2 was observed in 3.2%. We did not observe any AEs of grade 3, but we did observe some side effects (e.g., carotid artery dissection, pseudoaneurysm at the puncture site, epileptic seizures, and drowsiness), which were not reported by this researcher. As can be seen, the distribution of particular side effects in our study, Mikuni’s study, as well as other researchers’ papers vary, which is probably a result of slight differences in performing the Wada test between these centres. Further analyses of these differences could reveal what each of the centres need to improve to increase the safety of the procedure.

No prior reports on the AEs of the Wada procedure in Poland are available, and to the best of our knowledge, this study consisted of the largest sample of patients. We proposed dividing AEs into three groups: 1) major, demanding medical intervention and/or further medical assistance; 2) transient and mild, which did not require an additional intervention of a physician; and 3) epileptic seizures.

In our study, the rate of serious complications (group 1) during the Wada test was very low (1.6%), and they were related to the angiography procedure. This percentage is in line with studies assessing the number of AEs during standard angiography^20–22^. Alsallom^16^ reported one serious but not persistent AE related to an angiography procedure; i.e., a segmental ICA spasm, and Loddenkemper^19^ noted a case of carotid dissection during the Wada test with amobarbital.

In this study, transient and mild AEs occurred often (37.5% of cases), which is similar to Curot’s ^13^ observations, noting AEs in 44.8% cases of propofol injections. None of the patients developed further consequences or demanded additional medical assistance. The most common AEs observed in our study were eye pain, shivers, abnormal behavioural and emotional changes, as reported in other studies^13–18^. A red, painful eye can be the result of cross-filling or anastomosis between the internal carotid and external carotid arteries. Curot^13^ hypothesized that a slower injection of propofol would help to avoid the occurrence of this side effect. Shivers were also common among our patients. The mechanism of postanaesthetic shivers is explained as a result of thermoregulatory mechanisms due to action of general or regional anaesthetic agents, including propofol, which lead to inadvertent hypothermia^23–26^. Mikuni^17^ noted that shivers might also be a result of a drug-induced allergic reaction.

Disturbances of consciousness in this study were noted rather rarely and we counted them together with apathy, viscosity and verbosity as behavioural changes. We observed minor drowsiness only in two propofol injections (0.8%) and this did not affect the Wada procedure. In Curot’s^13^ study, the authors observed disturbances of consciousness in 12.1% of propofol injections, but they were mostly mild and of short duration. Other authors did not notice this type of AE. Mikati^14^ observed apathy in one patient, which was explained as the psychological effect of the anaesthetic agent.

Emotional changes appeared in our patients, mostly exhibited as laughter and a jocular attitude. Statistical analysis showed that laughter was observed more often after the injection in a dominant hemisphere (mostly left). This is an interesting result as many previous studies have shown that injecting an anaesthetic agent (in cited reports, it was amobarbital) into the right carotid artery caused a euphoric reaction, smiling, and the reverse, as injection to the left carotid artery, resulted in sadness and crying. Those studies led to the valance hypothesis stating that the left hemisphere is “positive” and the right hemisphere specializes in negative emotions^27^. In the studies of Curot^13^, Takayama^15^, Alsallom^16^, and Mikuni^17^ transient euphoria was observed, and the authors reported this adverse effect after the injection of propofol into both the left and right ICA. The appearance of laughter or euphoria can be explained by disinhibition of the frontal lobe function due to the injection of the anaesthetic or to the propofol molecule^13^. Additionally, as hypothesized by Mikuni^17^, it might be a psychological effect of the anaesthetic agent. This less typical finding in our study, i.e., that laugher dominates after propofol injection into the dominant hemisphere, can also be explained by the fact that many of our patients were amused by their slurred speech due to aphasia (they laughed after their failed attempts to speak), and only a small number of patients reacted with crying or sadness (15 vs 4 of 122 patients). We believe that patients are more likely to react with amusement to speech difficulties if they have been told in advance that they might experience trouble talking and that this is transient.

Mikuni^17^ observed increased muscle tone with twitching and rhythmic movements or tonic posture in 12% of propofol patients, which is mostly apparent among those patients who received the higher dose of propofol. We did not find similar complications, even in the group of patients who received more anaesthetic.

Our third group consisted of epileptic seizures (this happened twice). Both events were recognized as seizures as they were of the same appearance as typical attacks experienced by those patients on a regular basis. One event occurred before neuropsychological testing, during the angiography, and one occurred in the middle of the Wada test. There is evidence that receiving propofol might induce seizures and cause “seizure-like” phenomena^28^, but in our opinion, it was not the case in this study. Curot^13^ noted increased interictal epileptic activity in one patient, but no seizures. Mikati^14^ reported one case of a man who experienced combativeness, confusion and agitation, but those symptoms were distinct from his typical clinical seizures.

It is worth mentioning that all of the referred studies had some differences in the Wada procedure (the dose of propofol injected, speed of the injection, and uni- vs. bilateral examination), different types of brain lesions, different ways of classifying particular events as adverse and variable numbers of patients. These factors might influence or even prevent making clear and reliable comparisons.

## Conclusions

Despite many attempts to determine a less invasive way to assess hippocampal capacity and language lateralization in epilepsy patients, the Wada test is still used in some centres and is perceived to be an important component of presurgical diagnostics, when an fMRI cannot be performed. Because of the usefulness of this procedure, it is important to assess the potential risk of conducting the Wada testing with a particular anaesthetic. In our study, most of the AEs of the Wada test with propofol were short-lasting, mild and reversible. Only two grave AEs were observed due to the angiography procedure, the Wada test or the anaesthetic itself. In conclusion, the Wada test with the use of propofol carries a low risk of a serious health damage and the benefits acquired from running it exceed the losses. In our opinion, it can be perceived as a good method for assessing language and memory in some of the epilepsy surgery candidates, especially when fMRI is not available or feasible in a particular case.

## Methods

### Subjects

We retrospectively reviewed the medical charts of 122 consecutive patients with drug resistant epilepsy, who underwent the Wada test with propofol in the period of 2006 to 2016 as a part of the presurgical examination at the Department of Neurosurgery of the Medical University of Warsaw. The group consisted of sixty-six women and fifty-six men, aged seventeen years or older, with unilateral seizure onset zone. Patients with bilateral epileptogenic foci were not candidates for surgery, so they did not undergo the Wada procedure.

In 118 patients, the Wada test was performed bilaterally and, in four, due to complications that occurred in the first half - only unilaterally. To assess the number and type of potential adverse events, we decided to evaluate not only proportion of patients with AEs but also to assess each side of the procedure separately. Therefore, in total we analysed 240 cases. In 104 patients (204 cases), 10 mg of propofol was injected while 22 patients (36 cases) had a higher dose of propofol (extra 3–5 mg) due to the lack of a neurological effect of the drug (none or only slight contralateral hemiplegia), administered bilaterally or unilaterally.

The demographic and clinical characteristics of all subjects are presented in Table 5.

**Table 5:** Demographic and clinical characteristics of patients undergoing the Wada test.

		Seizure onset zone in the right hemisphere	Seizure onset zone in the left hemisphere
Number of patients		50	72
Male/Female		31/27	35/29
Age, mean years (SD)		30.8 (8.75)	32.6 (9.42)
Handedness		Right: 41; Left; 9; Ambidexterity: 0	Right: 66; Left; 4; Ambidexterity: 2
Age at epilepsy onset, mean years (SD)		12.99 (8.59)	13.63 (8.12)
Duration of the epilepsy, mean years (SD)		18.1 (9.78)	19.2 (9.03)
Types of lesions: tumour/cavernoma/ focal cortical dysplasia/focal cortical dysplasia & hippocampal sclerosis		0/3/18/29	3/4/29/36
Results of the Wada procedure	Hemispheric dominance for speech	Left: 41; Right: 1, Bilateral: 8	Left: 52; Right: 8, Bilateral: 12
	Contralateral hippocampus’ capacity for memory – number of patients for each range	<50%: 0 50–75%: 4 >75%: 46	<50%: 6 50–75%: 27 >75%: 39
	Ipsilateral hippocampus’ capacity for memory – number of patients for each range	<50%: 8 50–75%: 21 >75%: 19	<50%: 8 50–75%: 21 >75%: 41

Comments: capacity for memory was assessed as the % of recalled items; for 2 patients with right epileptogenic hemisphere and 2 with left epileptogenic hemisphere there were no possibility to assess ipsilateral hippocampus’s capacity due to panic attack.

### Wada test

Before the procedure, detailed information regarding its course was provided to the patient. The physician as well as neuropsychologist listed and explained the most common sensations that would be experienced by the patient, such as transient hemiparesis, aphasia, hemianopia, etc. Patients were encouraged to ask questions and all issues were discussed until everything was understood and accepted. As the first stage of the procedure, before each propofol injection, intracranial circulation angiography (via the transfemoral approach) was performed to exclude the existence of anomalies possibly changing the flow of the anaesthetic agent. In our study, we did not observe any serious anomalies, which would make the procedure unable to continue. The atypical anatomy of the vascular system - foetal-type posterior circle of Willis, was observed in 39 (32%) of the patients – in 15 bilaterally, in 12 left-sided, and in 12 right-sided (54 cases). Subsequently, the anaesthetic was administered by hand-push injection through the catheter into the internal carotid artery (ICA) to induce anaesthesia, first to the hemisphere considered as epileptogenic, and after 30 minutes’ interval, to the contralateral hemisphere.

The standard dose of propofol was 10 mg (1 ml) per injection into each ICA, dissolved in 9 ml of saline. The additional portion (3–5 mg) was immediately added when the neurologist stated a lack of contralateral hemiplegia. To administer propofol, a modified version of the Kyoto University Graduate School of Medicine Protocol^15^ was used: application of 2 ml of the solution slowly (approx. 10–15 s), with the remaining 8 ml applied as a bolus. In our experience, this method of drug administration reduced the emergence of possible side effects, especially psychomotor agitation and disturbances in consciousness.

Before administering the propofol injection, the patients were asked to put their hands up and count aloud. After injecting the whole portion of the anaesthetic solution, contralateral hemiplegia and hemifacial weakness were observed, a sign of effective anaesthesia (15 s from beginning the injection on average). The patient was asked to answer questions and react on commands to evaluate the level of logical contact essential for further assessment. Neuropsychological testing began when there was sufficient cooperation with the patient. In cases where aphasia precluded proper cooperation, the examination was deferred until it was possible for the patient to liaise. Therefore, the stimuli were shown after approximately 60 s for the dominant and 150 s for the non-dominant hemispheres. The memory evaluation consisted of seven components: two words, one sentence, two real objects and two simple questions the patient was expected to answer (e.g., “Where do you live?”). All stimuli were presented twice. After approximately 3 minutes from object presentation, free recall and recognition memory were tested. The neuropsychological evaluation was done for each hemisphere. The results of the Wada procedure for hemispheric speech dominance and memory are presented in Table 5.

All procedures were in accordance with the Declaration of Helsinki and approved by the Department of Psychology of the University of Warsaw Ethics Committee. All participants gave written informed consent. For one participant under the age of 18, informed consent was obtained from a parent.

### AEs classification

The AEs were classified into three groups: (1) major and severe, (2) mild and transient, and (3) epileptic seizures. Major, severe complications were related to the angiography procedure, lasted longer than the Wada test, carried a risk of permanent damage to health and required an additional medical intervention (in our research it was: a) artery dissection, which caused a small area of ischaemia in the left frontal lobe and transient aphasia, and b) pseudoaneurysm in the area of the femoral artery puncture). Mild and transient adverse events, were related to the procedure, passed with the completion of the anaesthetic action and did not constitute any threat or significant inconvenience for the patient. AEs from this group were divided into three subgroups. The first (Group 2a) included somatic symptoms: eye pain and/or lacrimation and shivers. The second subgroup (Group 2b) included behavioural abnormalities such as verbal disinhibition, viscosity (enhanced interpersonal adhesiveness, stickiness of thought)^29^, apathy and drowsiness. The third subgroup (Group 2c) included emotional lability such as sadness or uncontrollable laughter. Third group included epileptic seizures – undesirable events disrupting the course of the procedure, without a clear relation to the Wada test.

### Statistical analysis

Data were entered into SPSS v.21. Differences in the distributions of particular variables in adverse and non-adverse events groups were tested with the chi square test. The results were recognized as being statistically significant when p < 0.05.

## Data Availability

Data supporting the results are available by request. Please contact the corresponding author.
